# Supplementary material for: Myelin-reactive B cells exacerbate CD4+ T cell-driven CNS autoimmunity in an IL-23-dependent manner
Source: Nat Commun. 2024 Jun 26;15:5404. doi: 10.1038/s41467-024-49259-0 (PMC11208426; doi:10.1038/s41467-024-49259-0)
Supplement: Supplementary file 1 — Supplementary Information [file 41467_2024_49259_MOESM1_ESM.pdf]

Supplementary Figure 1.

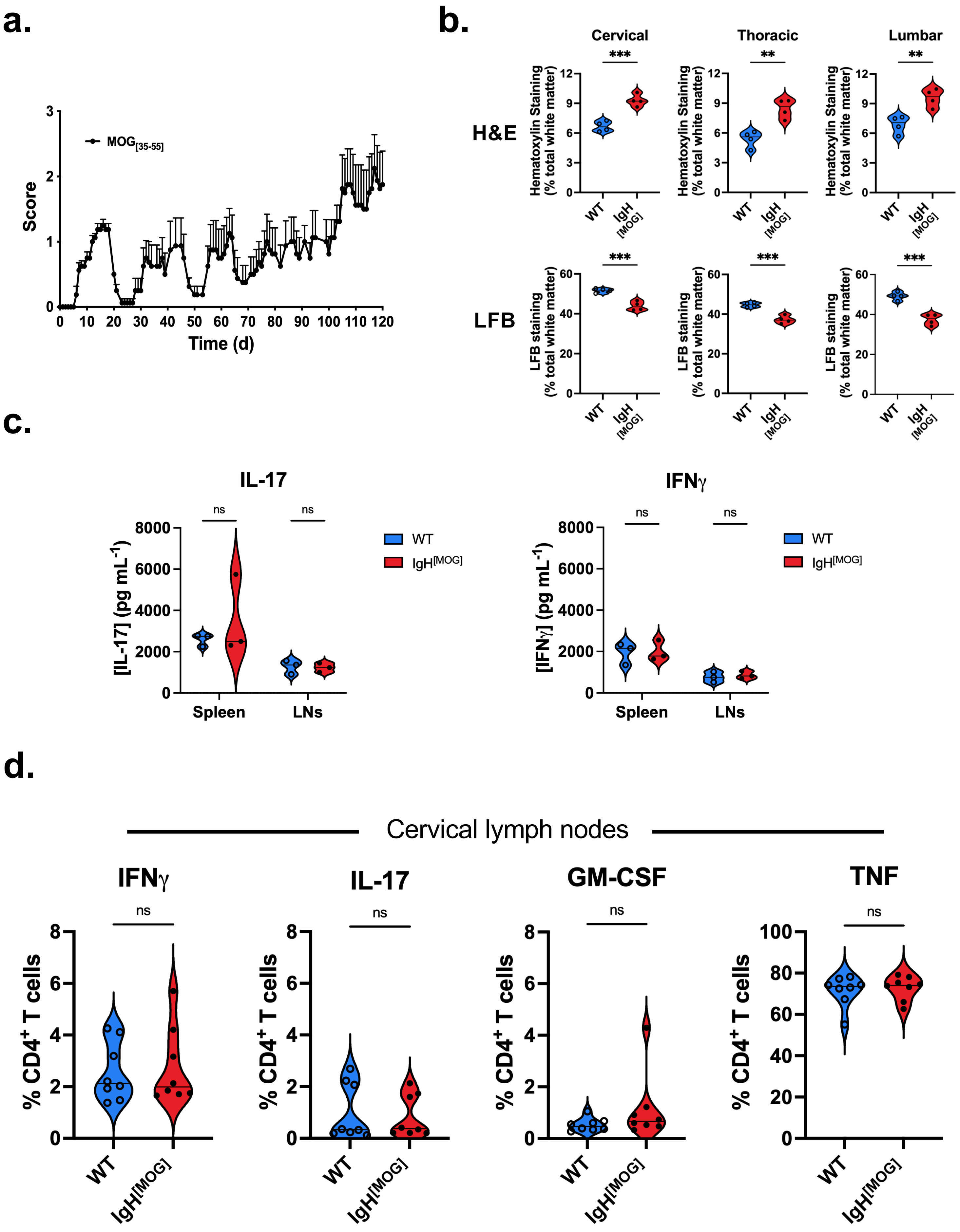

Supplementary Figure 1. Data linked to main Figure 1. a. Female WT NOD mice (n=8) were immunized with $MOG_{[35-55]}$ and were monitored for signs of EAE for 120 days. Data are from a single immunization. **b.** Quantification of H&E and LFB staining from experiment in which representative plots are shown in **Fig 1b.** Two-tailed *t*-test was used. **c.** Splenic and draining LN cells were isolated from female WT (n=3) and $IgH^{[MOG]}$ (n=3) cells at disease onset from a single immunization, and were stimulated with 10 μg mL^{-1} $MOG_{[35-55]}$. Supernatants were collected at d5 and IFNγ and IL-17 were assessed by ELISA. Tukey's post-hoc test after one-way ANOVA was used. **d.** Female WT (n=8) and $IgH^{[MOG]}$ (n=8) mice were immunized with $MOG_{[35-55]}$, and $CD4^+$ T cells were isolated from cervical LNs at endpoints and assessed for expression of the indicated cytokines. Data from two immunizations. Two-tailed *t*-test was used. In all violin plots, horizontal lines represent medians. Each datapoint represents an individual mouse. Exact p-values can be found in the Source Data file.

Supplementary Figure 2.

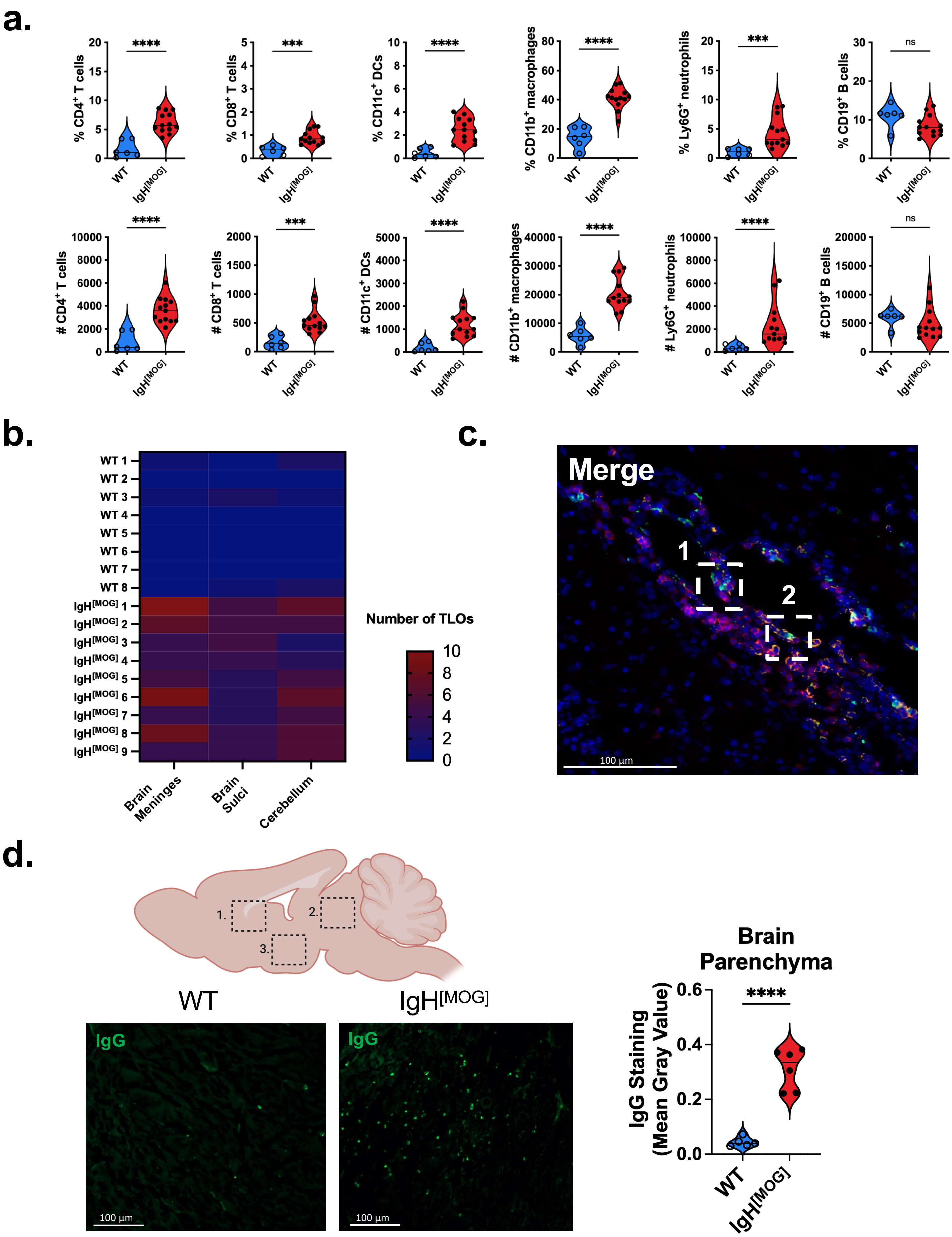

Supplementary Figure 2. Data linked to main Figure 2. a. Mononuclear cells were isolated from the CNS of immunized male WT (n=6) and IgH$^{[MOG]}$ (n=13) mice. Frequency and absolute numbers of CD19^{+} B cells, CD4^{+} and CD8^{+} T cells, Ly6G^{+} neutrophils, CD11b^{+}CD11c^{-} macrophages and CD11c^{+} dendritic cells were enumerated by flow cytometry. Data are representative of one of two immunizations. Two-tailed *t*-test was used. **b.** Heatmap depicting the distribution of lymphocytic aggregates (from mice in **Figure 2a**) in the brain meninges, brain sulci and cerebellar meninges. **c.** Regions of interest (dotted boxes) in the IgH$^{[MOG]}$ meningeal lymphocytic cluster from which the images in **Figure 2c** are derived. Region #1 corresponds to the top row of **Figure 2c** and #2 to the bottom row. B220 (red), CD138 (yellow) and AID (green). **d.** IgG staining was quantified from sulci-proximal (area 1), cerebellar meninges-proximal (area 2) and brain meninges-proximal (area 3) parenchymal tissue of WT (n=5) vs IgH$^{[MOG]}$ (n=6) female mice. Representative 20X images are from sulci-proximal tissue. Each data point represents the IgG positivity for an individual mouse, averaged over the 3 regions. Data are from a single immunization. Two-tailed *t*-test was used. In all violin plots, horizontal lines represent medians. Each datapoint represents an individual mouse. Exact p-values can be found in the Source Data file.

Supplementary Figure 3.

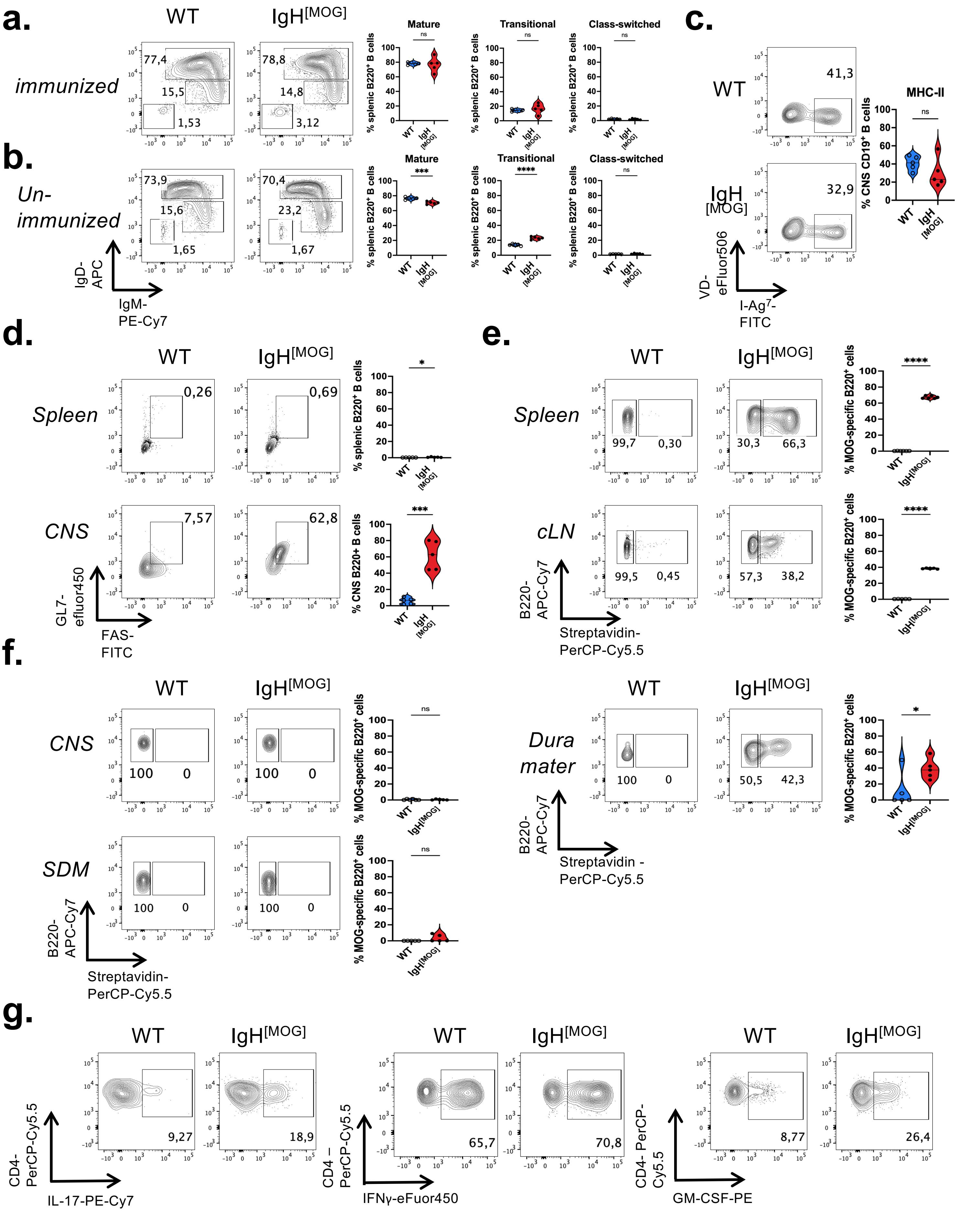

Supplementary Figure 3. Data linked to main Figure 3. a. Splenic mature, transitional and CS B cells were assessed from the same mice as in **Figure 3a**. Two-tailed *t*-test was used. **b**. Splenic mature, transitional and CS B cells were assessed from unimmunized female WT (n=5) and IgH$^{[MOG]}$ (n=5) mice. Data is from one experiment. Two-tailed *t*-test was used. **c.** MHC class II (I-Ag^{7}) expression was assessed from CNS B cells at endpoint from male WT (n=5) or IgH$^{[MOG]}$ (n=5) mice. Representative of one of two immunizations. Two-tailed *t*-test was used. **d.** Germinal center B cells (GL-7^{+}Fas^{+}) were assessed from the spleens and CNS of male WT (n=5) or IgH$^{[MOG]}$ (n=5) mice from a single immunization. Two-tailed *t*-test was used. **e, f.** MOG-specificity of B cells was assessed from the spleens and cervical LNs (**e**), and CNS, subdural enriched meninges and dura mater (**f**) of immunized female WT (n=5) and IgH$^{[MOG]}$ (n=5) mice. Single cell suspensions were labeled with biotinylated MOG followed by fluorescently labeled streptavidin. Representative of one of two immunizations. Two-tailed *t*-test was used. **g.** Representative plots for the data presented in **Figure 3e**. In all violin plots, horizontal lines represent medians. Each datapoint represents an individual mouse. Exact p-values can be found in the Source Data file.

Supplementary Figure 4.

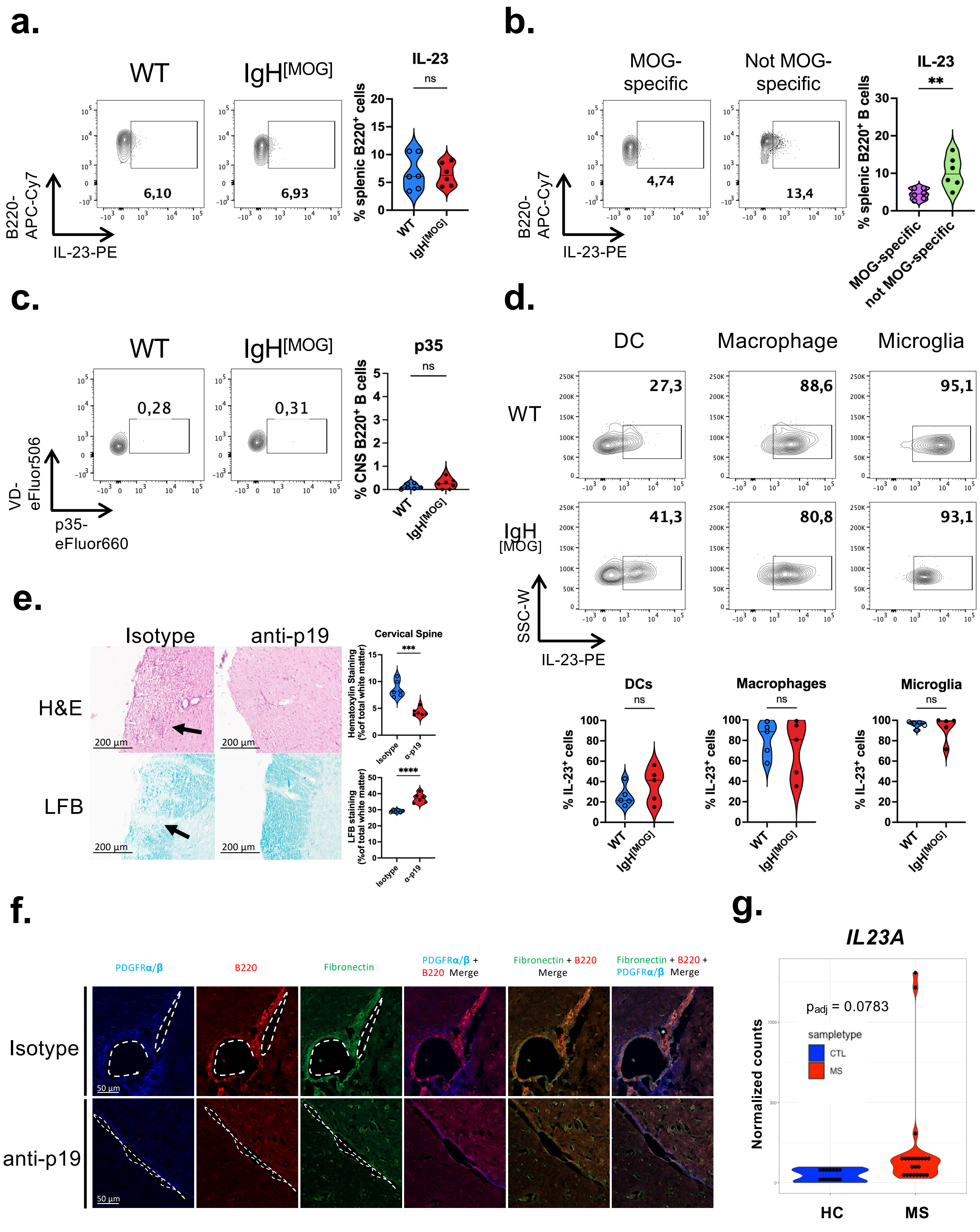

Supplementary Figure 4. Data linked to main Figure 4. a. Positivity of splenic B cells for IL-23 heterodimer expression was assessed from immunized female WT (n=6) and $IgH^{[MOG]}$ (n=6) mice. Data representative of one of four immunizations. Two-tailed *t*-test was used. **b.** B cells from $IgH^{[MOG]}$ mice in (**a**) were gated as MOG-nonspecific or MOG-specific and IL-23 expression was assessed from each population. Data representative of one of two immunizations. Two-tailed *t*-test was used. **c.** WT (n=6) and $IgH^{[MOG]}$ (n=7) mice were immunized with $MOG_{[35-55]}$ and B cells were isolated from CNS tissues at endpoints. Expression of IL-12p35 was assessed by intracellular flow cytometry. Data representative of one of two immunizations. Two-tailed *t*-test was used. **d.** Dendritic cells ($CD11c^+$), macrophages ($CD11b^+$), microglia ($CD45^{mid}CD11c^+CD11b^-$) were assessed for expression of IL-23 heterodimer from the CNS of male $MOG_{[35-55]}$-immunized WT (n=5) or $IgH^{[MOG]}$ (n=5). Data from one immunization. Two-tailed *t*-test was used. **e.** Inflammatory foci (H&E) and demyelination (LFB) were quantified from cervical spine of female $IgH^{[MOG]}$ mice from a single immunization, treated with anti-p19 (n=3) or rIgG1 isotype (n=3). Two-tailed *t*-test was used. **f.** ECM deposition underneath meningeal lymphoid aggregates was identified from the samples in **Figure 4d**. **g.** *hIL23A* transcript expression was assessed from B cells of 23 female pwMS and 14 female age-matched healthy controls (HC). Transcriptomes derived from GEO series GSE173789. *t*-test with Benjamini-Hochberg correction applied. In all violin plots, horizontal lines represent medians. Each datapoint represents an individual mouse. Exact p-values can be found in the Source Data file.

Supplementary Figure 5.

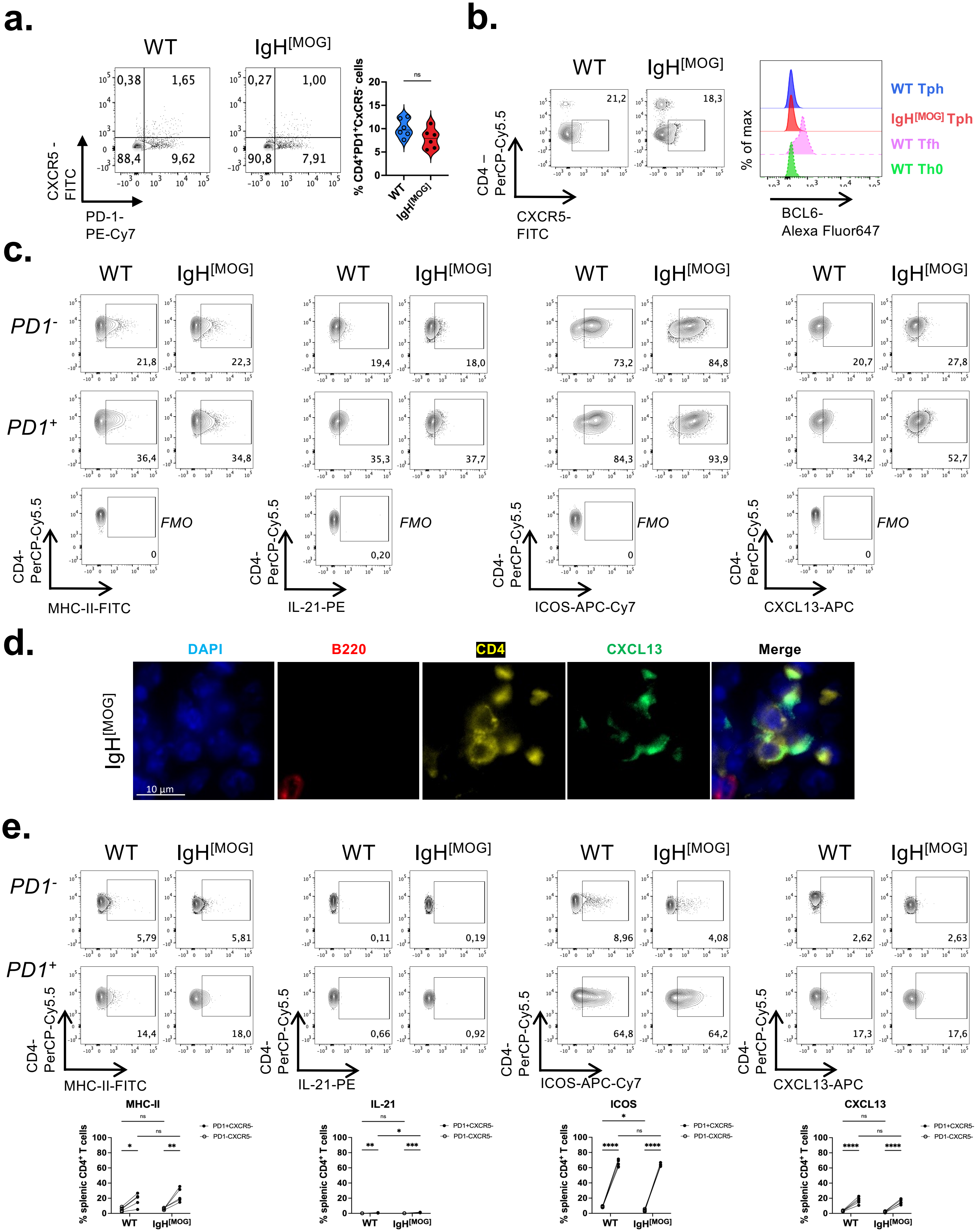

Supplementary Figure 5. Data linked to main Figure 5. a. Splenic $CD4^+$ T cells were assessed for expression of PD-1 and CXCR5 from the same mice as in **Figure 5a**. Two-tailed *t*-test was used. **b.** *Left:* Representative plots depicting $CD4^{neg}CXCR5^+$ cells in the CNS of WT and $IgH^{[MOG]}$ immunized mice at endpoint. Gates set on FMO. *Right:* Expression of Bcl6 was assessed from female WT or $IgH^{[MOG]}$ PD-1^+CXCR5^- CNS-infiltrating T cells versus in vitro generated 1C6 Tfh or Th0. Data is from one experiment **c.** Representative flow cytometry plots and FMOs of CNS T cells corresponding to quantification in **Figure 5c**. Live $CD4^+$ pre-gate was applied. Marker gates were based on fluorescence minus one (FMO) controls from CNS. **d.** IF image of CXCL13 (green) and CD4 (yellow) colocalization in the meninges of an immunized $IgH^{[MOG]}$ mouse. B220, red; DAPI, blue. Representative of 4 female mice from a single immunization. **e.** Expression of MHC class II, IL-21, ICOS and CXCL13 was assessed from splenic $CD4^+PD1^+$ and PD-1^{neg} T cells from the same immunized WT and $IgH^{[MOG]}$ mice as in **Figure 5c**. Live $CD4^+$ pre-gate was applied. Marker gates were based on FMO controls. Sidak's multiple comparisons test after repeated measures two-way ANOVA was used. In all violin plots, horizontal lines represent medians. Each datapoint represents an individual mouse. Datapoints connected by a line represent paired observations from the same mouse. Exact p-values can be found in the Source Data file.

Supplementary Figure 6.

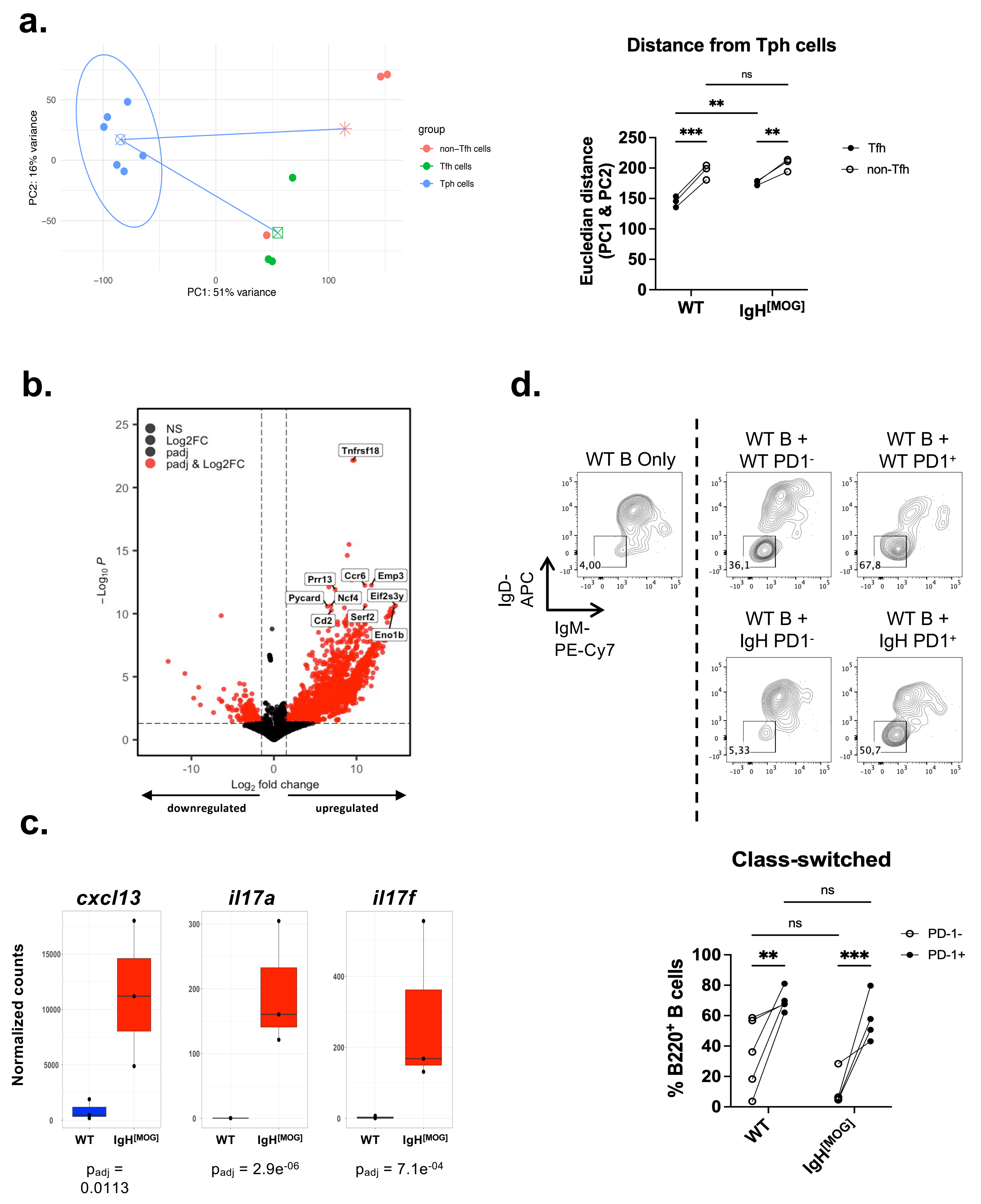

Supplementary Figure 6. Data linked to main Figure 5. a. Euclidean distance between the transcriptome of CNS-infiltrating WT and IgH$^{[MOG]}$ CD4^{+}PD1^{+}CXCR5^{-} cells (n=3) and the centroids of Tfh and non-Tfh samples from GSE181433. Lines in the PCA plot depict the distance between the centroid of all CD4^{+}PD1^{+}CXCR5^{-} transcriptomes and the centroids of Tfh vs non-Tfh cells. Quantification represents Euclidean distance from each WT or IgH$^{[MOG]}$ transcriptome to the centroid of the Tfh or non-Tfh population in GSE181433. Sidak's multiple comparisons test after repeated measures two-way ANOVA was used. **b.** Volcano plot of mRNA-seq data depicting DEG (1.5x-fold increase, p_{adj}<0.05) between WT (n=3) and IgH$^{[MOG]}$ (n=3) CNS-infiltrating PD1^{+}CXCR5^{-}CD4^{+} T cells. The top 10 DEG are labeled on the plot. **c.** Normalized counts for *Cxcl13*, *Il17a* and *Il17f* between CNS-infiltrating WT and IgH$^{[MOG]}$ CD4^{+}PD-1^{+}CXCR5^{-} T cells. *t*-test, with Benjamini-Hochberg correction applied (p_{adj}). **d.** WT B cells were cultured at a 1:1 ratio with splenic PD1^{+}CXCR5^{-} or PD1^{neg} CD4^{+} T cells from immunized male WT (n=5) or IgH$^{[MOG]}$ (n=5) mice. After 5 days, B cells were assessed for expression of IgM and IgD. Gated on live B220^{+} events. Representative of one of two immunizations. Sidak's multiple comparisons test after repeated measures two-way ANOVA was used. In all violin plots, horizontal lines represent medians. In box and whisker plots, whiskers represent range and horizontal lines represent medians. Each datapoint represents an individual mouse. Datapoints connected by a line represent paired observations from the same mouse. Exact p-values can be found in the Source Data file.

Supplementary Figure 7.

a.

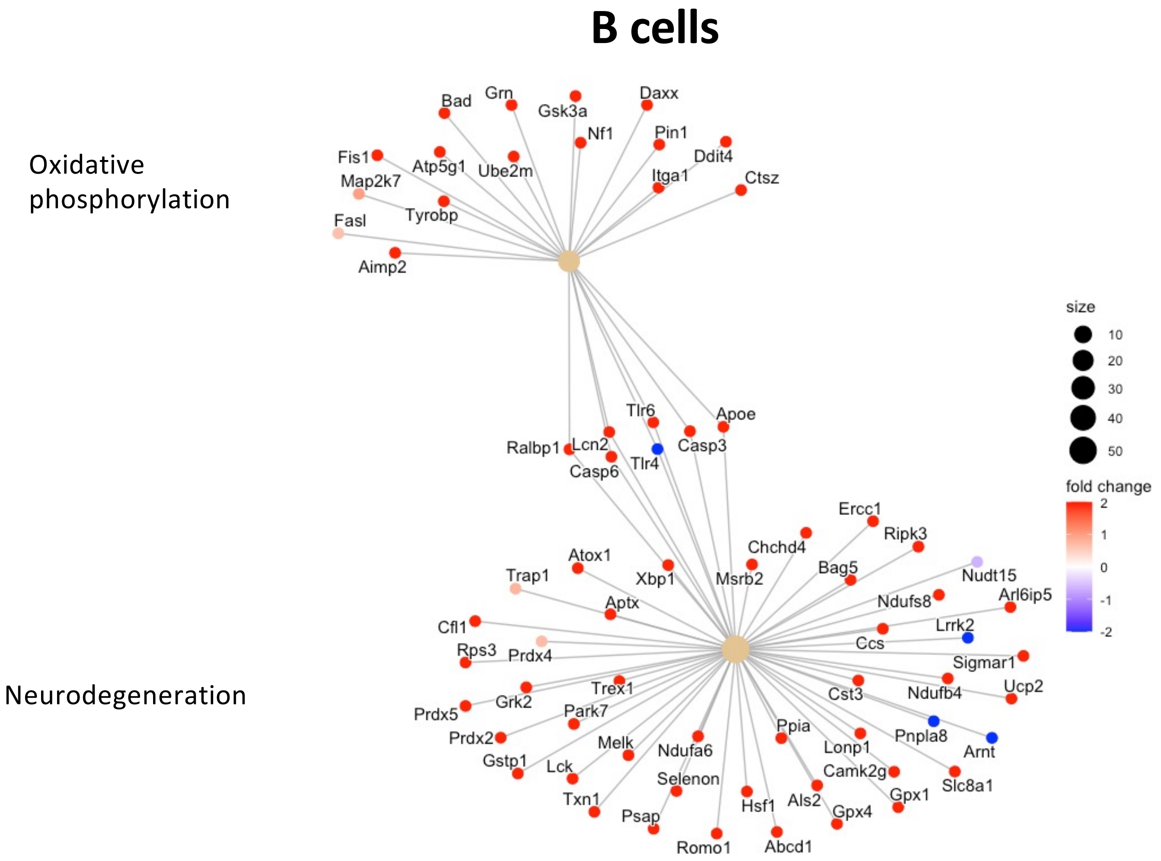

b.

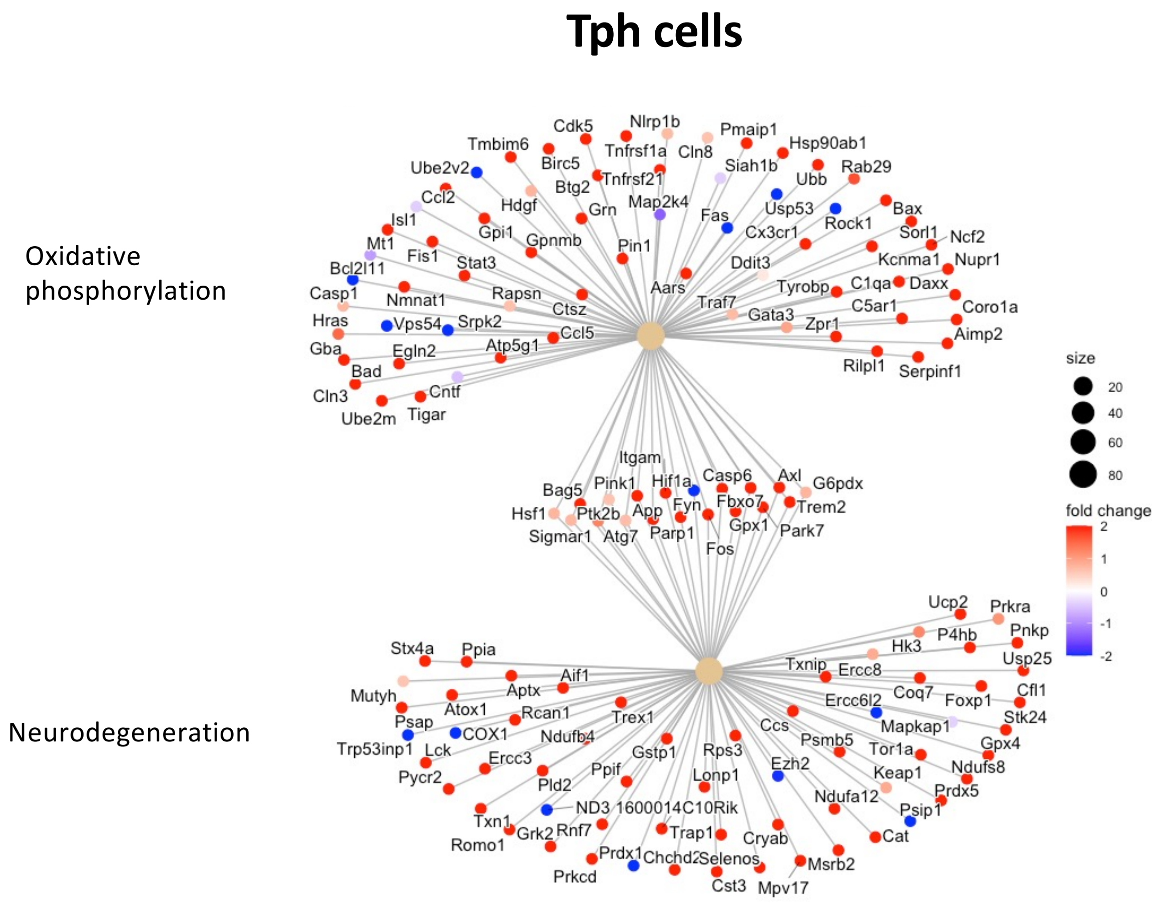

Supplementary Figure 7. Data linked to main Figure 6. CNET plots depicting relationships between DEGs, associated with the terms “oxidative phosphorylation” and “pathways of neurodegeneration”, between WT and $IgH^{[MOG]}$ CNS-infiltrating B (**a**) and Tph-like (**b**) cells. All DEGs were p_{adj} <0.05.

Supplementary Table 1.

Diagnosis	Sex	Disease modifying therapy	Steroids
RRMS	F	No	No
RRMS	F	No	No
RRMS	M	No	No
RRMS	F	Tysabri (natalizumab)	Yes
RRMS	F	Tysabri	No
RRMS	F	Tysabri	No
RRMS	F	No	No
RRMS	F	No	No
RRMS	F	Tecfidera (dimethyl fumarate)	No
RRMS	M	No	No
PPMS	F	No	No

Supplementary Table 1. Characteristics of people with MS studied in main Figure 4g. RRMS, relapse-remitting MS; PPMS, primary progressive MS; F, female; M, male. Age range, 31-56 years.

Representative gating summary

Gating strategy for B cells and CD4+ T cells in the CNS for different cytokines

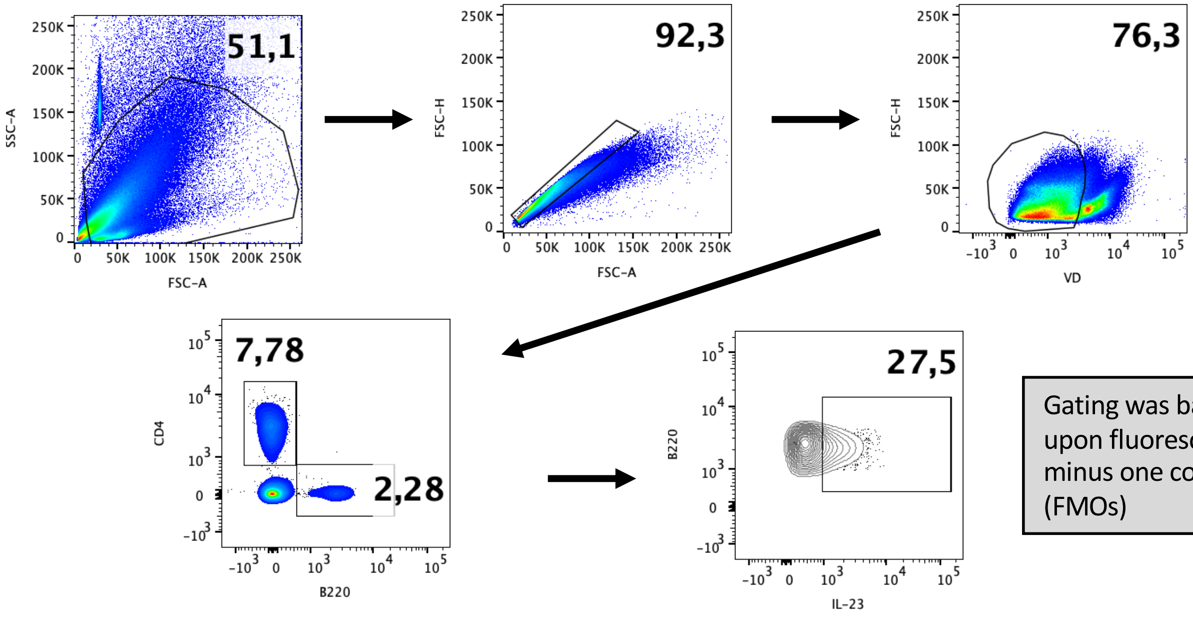

Gating was based upon fluorescence minus one controls (FMOs)

Gating strategy for Tph cells in the dura mater

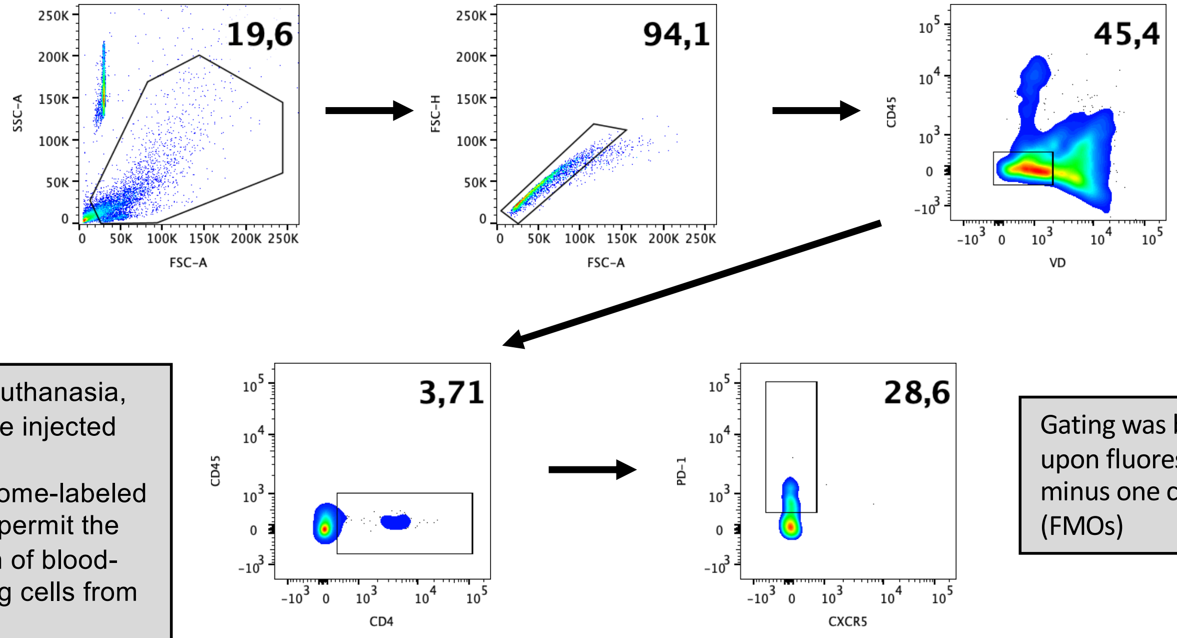

Prior to euthanasia, mice were injected i.v. with fluorochrome-labeled CD45 to permit the exclusion of blood-circulating cells from analysis

Gating was based upon fluorescence minus one controls (FMOs)
